# Reliability and reproducibility of sciatic nerve magnetization transfer imaging and T2 relaxometry

**DOI:** 10.1007/s00330-021-08072-9

**Published:** 2021-06-09

**Authors:** Fabian Preisner, Rouven Behnisch, Olivia Foesleitner, Daniel Schwarz, Michaela Wehrstein, Hagen Meredig, Birgit Friedmann-Bette, Sabine Heiland, Martin Bendszus, Moritz Kronlage

**Affiliations:** 1 Department of Neuroradiology, Heidelberg University Hospital, Im Neuenheimer Feld 400, 69120 Heidelberg, Germany; 2 Institute of Medical Biometry and Informatics, University of Heidelberg, Im Neuenheimer Feld 130.3, 69120 Heidelberg, Germany; 3 Department of Sports Medicine (Internal Medicine VII), Medical Clinic, Heidelberg University Hospital, Im Neuenheimer Feld 410, 69120 Heidelberg, Germany

**Keywords:** Magnetic resonance imaging, Peripheral nervous system, Biomarkers, Observer variation, Reproducibility of results

## Abstract

**Objectives:**

To assess the interreader and test-retest reliability of magnetization transfer imaging (MTI) and T2 relaxometry in sciatic nerve MR neurography (MRN).

**Materials and methods:**

In this prospective study, 21 healthy volunteers were examined three times on separate days by a standardized MRN protocol at 3 Tesla, consisting of an MTI sequence, a multi-echo T2 relaxometry sequence, and a high-resolution T2-weighted sequence. Magnetization transfer ratio (MTR), T2 relaxation time, and proton spin density (PSD) of the sciatic nerve were assessed by two independent observers, and both interreader and test-retest reliability for all readout parameters were reported by intraclass correlation coefficients (ICCs) and standard error of measurement (SEM).

**Results:**

For the sciatic nerve, overall mean ± standard deviation MTR was 26.75 ± 3.5%, T2 was 64.54 ± 8.2 ms, and PSD was 340.93 ± 78.8. ICCs ranged between 0.81 (MTR) and 0.94 (PSD) for interreader reliability and between 0.75 (MTR) and 0.94 (PSD) for test-retest reliability. SEM for interreader reliability was 1.7% for MTR, 2.67 ms for T2, and 21.3 for PSD. SEM for test-retest reliability was 1.7% for MTR, 2.66 ms for T2, and 20.1 for PSD.

**Conclusions:**

MTI and T2 relaxometry of the sciatic nerve are reliable and reproducible. The values of measurement imprecision reported here may serve as a guide for correct interpretation of quantitative MRN biomarkers in future studies.

**Key Points:**

*• Magnetization transfer imaging (MTI) and T2 relaxometry of the sciatic nerve are reliable and reproducible.*

*• The imprecision that is unavoidably associated with different scans or different readers can be estimated by the here presented SEM values for the biomarkers T2, PSD, and MTR.*

*• These values may serve as a guide for correct interpretation of quantitative MRN biomarkers in future studies and possible clinical applications.*

**Supplementary Information:**

The online version contains supplementary material available at 10.1007/s00330-021-08072-9.

## Introduction

High-resolution magnetic resonance neurography (MRN) has emerged to a useful diagnostic tool for various neuropathies and allows detecting minor damage of the peripheral nervous system (PNS) with high sensitivity [1–6]. In a clinical setting, MRN is typically based on visual assessment of nerve lesions using high-resolution T2-weighted sequences.

Morphological nerve imaging can be complemented by quantitative MRI techniques, such as diffusion tensor imaging (DTI), which are increasingly studied in traumatic, hereditary, inflammatory, and degenerative neuropathies [7–9]. These techniques offer additional information about nerve microstructure or tissue composition and provide quantitative biomarkers. In addition to DTI—which has been well evaluated [10]—T2 relaxometry and magnetization transfer imaging (MTI) have increasingly been applied as novel quantitative MRN techniques in recent investigations [11–14].

T2 relaxometry yields the readout parameters transverse relaxation time (T2) and proton spin density (PSD) and is conducted by a multi-echo sequence and fitting of an exponential function. MR signal loss after a radiofrequency pulse usually follows an exponential decay characterized by the tissue-specific time constant T2, which describes the time in which the transverse magnetization decreases to 37% (1/e) of its initial value [15, 16]. PSD is another tissue-intrinsic parameter and refers to the concentration of protons excitable by MRI [17]. It equals the theoretical MR-signal-intensity without any effects of transverse relaxation. While T2 is considered a biomarker of free water, PSD is regarded to reflect total water content including protons bound to macromolecules such as myelin [16–18].

MTI is an MRI technique that generates the readout parameter magnetization transfer ratio (MTR) [19–21]. It relies on the principle that protons bound to macromolecules may be selectively saturated by an off-resonance radiofrequency pulse since resonance occurs in a larger bandwidth off the Larmor frequency in these bound protons compared to free-water protons. Magnetic saturation is then transferred to free-water protons which leads to a lower MR signal in a sequence with a saturation pulse than without. MTI works by a pair of MR sequences, one with and one without a preceding off-resonance saturation pulse. By calculating the relative difference between signals, the MTR can be determined, which reflects the concentration of bound protons and their interaction with protons in free water and is considered a biomarker of demyelination [22, 23].

Recent studies suggested that T2 relaxometry and MTI could yield promising MRN biomarkers for the assessment of various neuropathies [24–27]. However, these techniques have not been implemented into clinical routine yet. To use these parameters for decisions in individual patients, it is crucial to assess reliability by a quantitative estimation of the measurement error attributed to different examinations (test-retest) or different readers (interreader). First studies implemented reliability analyses of these techniques in their investigations with promising results, but a systematic assessment of both test-retest and interreader reliability in a larger cohort and a quantification of the measurement error are still lacking [11, 28, 29].

Reliability is commonly expressed by the intraclass correlation coefficient (ICC), a dimensionless parameter ranging between 0 and 1 [30]. However, the ICC should be interpreted cautiously since it depends not only on the measurement error but also on the sample variance. To describe measurement imprecision independently of the sample variance, the standard error of measurement (SEM) may be calculated, which indicates how test results spread around a “true” value [31, 32]. Besides, the SEM allows calculating the minimum detectable difference (MDD), which is the smallest difference needed between separate measurements in order for the difference to be considered real [31, 32]. Although sometimes sharing the same abbreviation, the standard error of measurement should not be confused with the standard error of the mean, which is considered a different statistical parameter.

The aim of the present study was to systematically assess interreader and test-retest reliability of MTI and T2 relaxometry in MRN and to quantify the measurement accuracy by means of ICC, SEM, and MDD. Being the most commonly examined and well-accessible nerve, we focused on the sciatic nerve and examined a cohort of 21 healthy participants who underwent three MRN examinations on separate days which were analyzed by two readers independently.

## Materials and methods

**Fig. 1:**
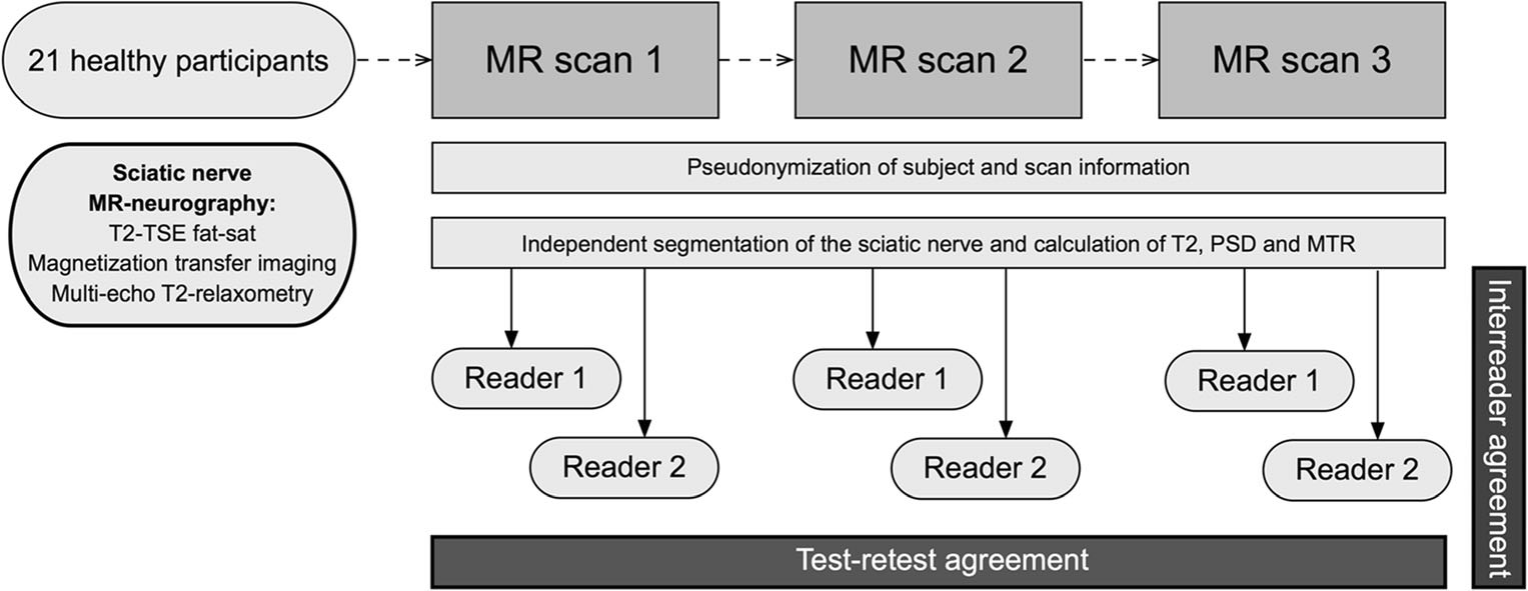
Study design flowchart. Twenty-one healthy participants underwent repeated MR neurography of their sciatic nerve on three separate days, each covering the exact same anatomical region. Image analysis and postprocessing were performed by two independent readers. Interreader agreement and test-retest reproducibility were statistically assessed for each of the biomarkers transverse relaxation time (T2), proton spin density (PSD), and magnetization transfer ratio (MTR)

This prospective study was approved by the institutional ethics board and written informed consent was obtained from all participants prior to the examinations. The study design is summarized in Fig. 1.

### Study subjects

Twenty-one healthy adult (>18 years) male volunteers were prospectively enrolled over a period of 22 months. Mean age was 24.1 ± 3 years (range: 20–30 years), mean weight was 76.8 ± 6.7 kg, mean height was 1.81 ± 0.1 m, and mean body mass index was 23.4 ± 1.7 kg/m^2^. Exclusion criteria were history of peripheral nerve disorders and general contraindications concerning MRI.

### MR data acquisition

All participants were prospectively examined at a 3-Tesla MR scanner (Magnetom Prisma-FIT, Siemens Healthineers) on three separate days using a 15-channel transmit-receive knee coil (Siemens Healthineers). The mean timespan was 5.7 ± 2.7 days between scans 1 and 2 and 4.8 ± 0.8 days between scans 2 and 3. MRN of the sciatic nerve of the dominant leg was conducted at mid to distal thigh level according to the following protocol (MR sequence parameters in Table 1):

1. An axial T2-weighted 2-dimensional turbo spin-echo (TSE) sequence to provide anatomical coverage with a high structural resolution,
2. An axial 2-dimensional multi-spin-echo (MSE) sequence for T2 relaxometry, and
3. Two-axial proton density–weighted 3-dimensional gradient-echo (GRE) FLASH sequences with and without an off-resonance saturation pulse (Gaussian envelop, duration 9984 μs, frequency offset 1200 Hz) at the exact same slice position for MTI. An adaptive inline image filter (Siemens Healthineers) was applied to reduce possible effects of B1 inhomogeneities on the received signal.

**Table 1:** MRN sequence parameters

	T2 TSE	T2 relaxometry	Magnetization transfer imaging
TR/TE (ms)	8640/54	2400/10, 20, 30 … 120	61/4.92
FOV (mm^2^)	160 × 160	160 × 160	160 × 160
Matrix size	512 × 333	192 × 169	128 ×128
Slice thickness (mm)	3.5	3.5	4
Interslice gap (mm)	0.35	0.35	-
No. of slices	41	13	18
Flip angle (°)	150	180	7
Fat saturation	Yes (spectral)	Yes (spectral)	Yes (spectral)
Acquisition time	6 min 48 s	6 min 50 s	1 min 56 s each (on + off)

### Image postprocessing

**Fig. 2:**
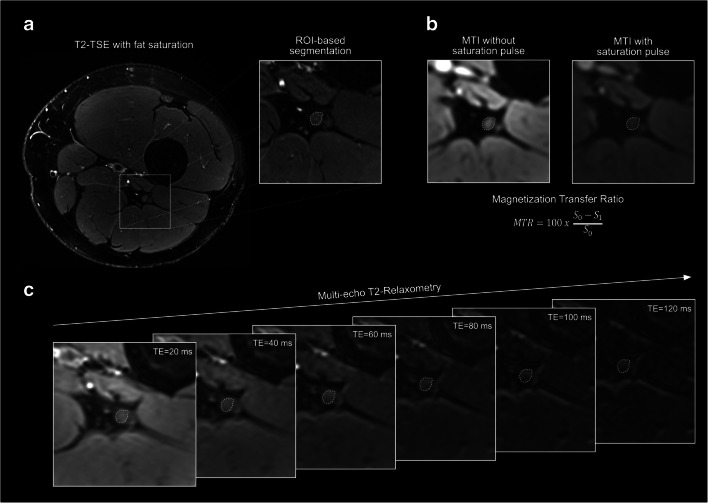
Image postprocessing. Nerve segmentation was performed by each reader independently by delineating the tibial portion of the sciatic nerve using the freehand region of interest (ROI) tool in Osirix in the T2-turbo spin-echo (TSE) sequence (**a**). Subsequently, ROIs were transferred onto co-registered gradient-echo sequences for magnetization transfer imaging (MTI) (**b**), and multi-echo sequences for T2-relaxometry (**c**) and quantitative readout parameters were assessed. MTR = magnetization transfer ratio, TE = echo time, S_0_ = MR signal intensity without saturation pulse, S_1_ = MR signal intensity with saturation pulse

Image postprocessing and segmentation were performed within 3 weeks after completion of image acquisition. First, images were visually assessed for motion artifacts and other artifacts that might impede segmentation of the sciatic nerve or that might modify readout parameters by projection on nervous tissue. All images were analyzed by two readers M.K. and F.P. with more than 6 and 4 years of experience in neuromuscular imaging, respectively, using the DICOM-viewer OsiriX (Pixmeo Sarl). Six central slices within each image slab covering the same anatomical region were identified by F.P. prior to further analysis. Manual nerve segmentation was subsequently performed in the high-resolution T2-weighted images by both readers independently and was restricted to the tibial portion of the sciatic nerve to prevent inclusion of paraneurial fat (for representative examples of image postprocessing, see Fig. 2) [33].

### T2 relaxometry

T2 relaxometry was based on MR signal analysis of the sciatic nerve in the MSE sequence. ROIs from the T2-weighted images were copied onto the corresponding MSE slice with TE = 20 ms, in which the boundaries of the sciatic nerve could be delineated best. Manual correction of distortion artifacts was performed by each reader independently, if necessary. Signal intensity for the whole ROI was determined for every echo time using the OsiriX plugin ROI enhancement, since averaging over all voxels helps to denoise data. Then, an exponential function was fitted:

$$ S(TE)=\mathrm{PSD}\times {\mathrm{e}}^{-\frac{TE}{T2}}+\mathrm{offset}, $$

as described previously [26, 33, 34], where S(TE) stands for the signal intensity at a given echo time TE, PSD is a dimensionless value for proton spin density, and T2 is the transverse relaxation time. To minimize systematic error, we restricted the analysis to the even echoes and used the offset as a fitting parameter, as described previously [34–36]. Additionally, a normalized PSD was calculated by dividing the PSD_nerve_ by a PSD of skeletal muscle (PSD_nerve_/PSD_muscle_). The ROI for measurement of PSD_muscle_ was placed in muscle tissue medially adjacent to the sciatic nerve (M. semimembranosus or adductor magnus). After slice-wise calculation of T2 and PSD, parameters were averaged from all six slices for further analysis.

### Magnetization transfer ratio

MTR was calculated based on sciatic nerve signal intensity in the pair of GRE sequences without (S_0_) and with (S_1_) off-resonance saturation pulse using the equation:

$$ \mathrm{MTR}=100\times \frac{\left({S}_0-{S}_1\right)}{S_0} $$

Analogously to T2 relaxometry, segmentation information was derived from the high-resolution T2 sequence. Likewise, signal intensity was averaged over all voxels to reduce possible effects of noise. If necessary, ROIs were manually adjusted for distortion or motion artifacts by both readers independently using the images without the off-resonance pulse. Additional computational co-registration between the two MTI sequences was not applied [37], as we were able to ascertain visually that ROIs aligned well with the nerve contours in both sequences (Fig. 2B). Subsequently, MTR was calculated for every slice and then averaged over all six slices.

### Statistical analysis

All values are shown as mean ± standard deviation. *p* values ≤ 0.05 were regarded as statistically significant. Statistical analyses were performed using SPSS (Version 24; SPSS Inc.) or R (Version 4.0.3; R Foundation for Statistical Computing). Graphs were created using GraphPad Prism (Version 8.3; GraphPad Software Inc.).

A single measurement, absolute agreement, two-way random effects model, ICC (2,1) according to Shrout and Fleiss was applied to assess interreader agreement and to calculate ICCs with 95% confidence intervals (CIs). To estimate test-retest reliability, a single measurement, absolute agreement, two-way mixed effects model was applied to calculate ICC (3,1) for each observer separately, and CIs were calculated accordingly. ICC values between 0.5 and 0.75, between 0.75 and 0.9, and greater 0.9 were regarded as moderate, good, and excellent agreement [30]. In addition, a Bland-Altmann analysis for repeated measurements was conducted and illustrated in Bland-Altmann plots. To assess measurement accuracy between readers and scans, SEM was calculated according to Popovic and Thomas [31]. MDD values for a CI of 95% were calculated according to the formula MDD = SEM x 1.96 x √2 [31, 32].

## Results

**Fig. 3:**
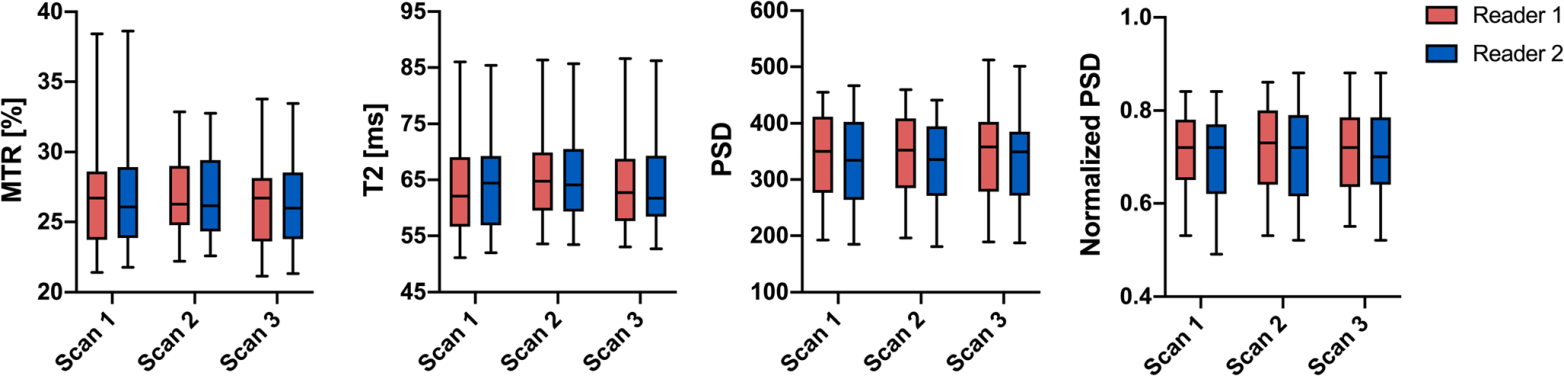
Descriptive statistics for both readers and all three scans. Values are illustrated as boxplots with whiskers to visualize measurement distribution (box showing the 25th to 75th percentiles with a line at the median, whiskers indicate the minimum and maximum data values). MTR = magnetization transfer ratio, T2 = transverse relaxation time, PSD = proton spin density

For the sciatic nerve, overall mean ± standard deviation MTR was 26.75 ± 3.5%, mean T2 relaxation time was 64.54 ± 8.2 ms, and mean PSD was 340.93 ± 78.8. Overall normalized PSD was 0.71 ± 0.09. Detailed descriptive statistics for every parameter, all three scans, and both readers are presented in Table 2 and Fig. 3.

**Table 2:** Descriptive statistics of multiparametric sciatic nerve MRN

	Scan 1		Scan 2		Scan 3	
	Reader 1	Reader 2	Reader 1	Reader 2	Reader 1	Reader 2
MTR (%)	26.8 ± 4.2	26.84 ± 4.2	26.97 ± 3.0	27.03 ± 3.0	26.35 ± 3.4	26.52 ± 3.3
T2 (ms)	63.93 ± 8.7	64.28 ± 8.6	65.57 ± 8.4	65.72 ± 8.3	63.66 ± 8.1	64.1 ± 8.1
PSD	344.3 ± 78.9	334.7 ± 82.7	341.7 ± 74.2	331.6 ± 76.5	351.9 ± 84.2	341.4 ± 84
Normalized PSD	0.7 ± 0.09	0.69 ± 0.1	0.71 ± 0.09	0.71 ± 0.1	0.71 ± 0.09	0.71 ± 0.09
CSA (mm^2^)	18.1 ± 4.5	18.9 ± 4.4	18.4 ± 4.6	19.1 ± 4.6	18.4 ± 4.6	18.7 ± 4.9

All values represent mean ± standard deviation. *MTR* magnetization transfer ratio, *T2* transverse relaxation time, *PSD* proton spin density, *CSA* cross-sectional area

Assessment of interreader reliability overall resulted in ICC values between 0.81 (for MTR) and 0.94 (for PSD), implying good to excellent agreement according to Koo and Li [30]. Calculation of test-retest reliability resulted in slightly lower ICCs ranging from 0.75 to 0.94. Lowest ICCs were observed for nerve MTR and highest for nerve PSD, respectively.

SEM for MTR was 1.7% for both interreader and test-retest agreement, SEM for T2 was 2.67 ms for interreader and 2.66 ms for test-retest agreement, and SEM for PSD was 21.3 and 20.1 for interreader and test-retest agreement, respectively. ICC, SEM, and MDD values for all parameters are listed in Table 3.

**Table 3:** Intraclass correlation coefficients (ICCs), standard error of measurement (SEM), and minimum detectable difference (MDD) values for interreader reliability and test-retest reliability

	Interreader ICC	Test-retest ICC		SEM		MDD	
		Reader 1	Reader 2	Interreader	Test-retest	Interreader	Test-retest
MTR	0.81 (0.60–0.92)	0.79 (0.62–0.90)	0.75 (0.55–0.88)	1.7%	1.7%	4.7%	4.7%
T2	0.92 (0.81–0.97)	0.92 (0.84–0.96)	0.9 (0.80–0.95)	2.67 ms	2.66 ms	7.4 ms	7.37 ms
PSD	0.94 (0.85–0.98)	0.94 (0.87–0.97)	0.94 (0.88–0.97)	21.3	20.1	59	55.7
Normalized PSD	0.89 (0.74–0.95)	0.9 (0.81–0.96)	0.89 (0.78–0.95)	0.03	0.03	0.08	0.08

Intraclass correlation coefficients (ICCs) with 95% confidence-interval were calculated according to Shrout and Fleiss. SEM and MDD calculations were based on Popovic and Thomas [31]. *MTR* magnetization transfer ratio, *T2* transverse relaxation time, *PSD* proton spin density

**Fig. 4:**
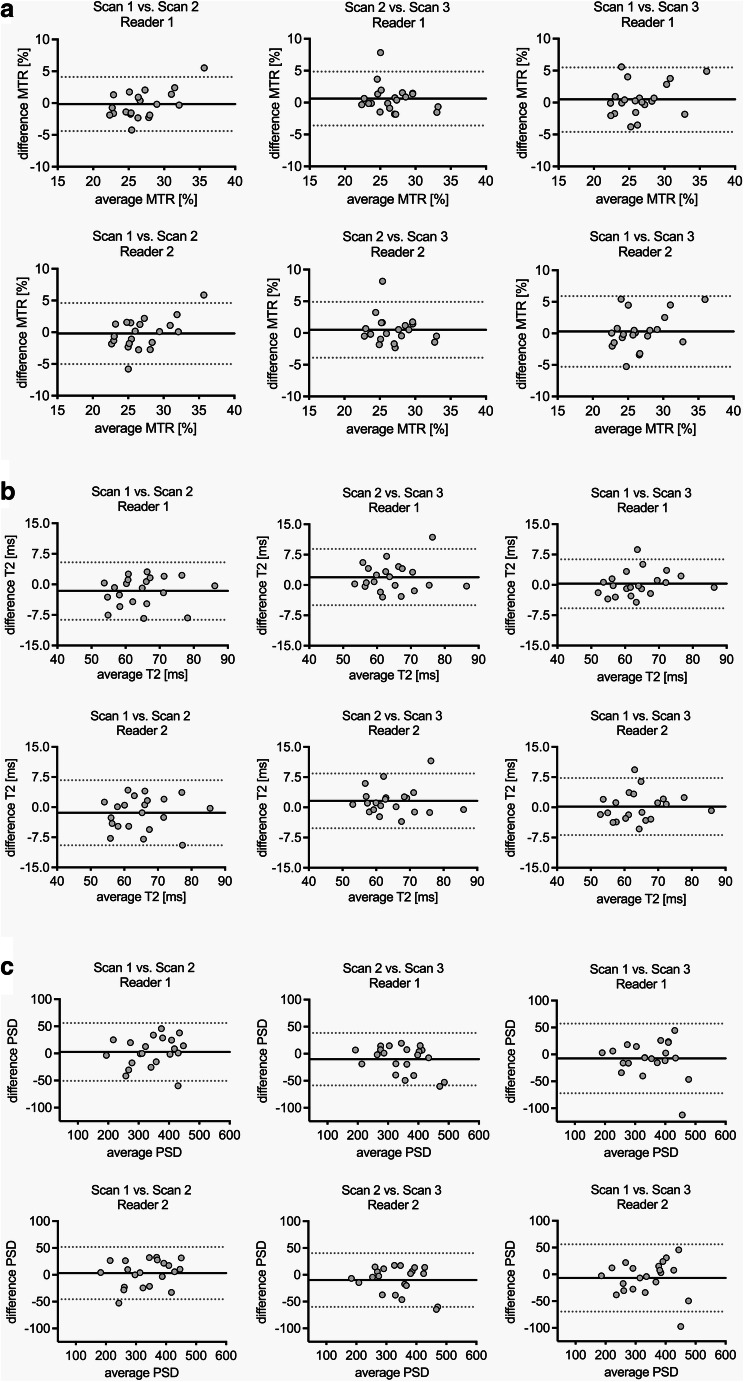
Bland-Altman plots for assessment of test-retest reliability of magnetization transfer ratio (MTR) (**a**), transverse relaxation time T2 (**b**), and proton spin density (PSD) (**c**) for both observers and all three scans, respectively. The black continuous line represents the mean of all differences (bias), grey dotted lines show the 95% limits of agreement

**Fig. 5:**
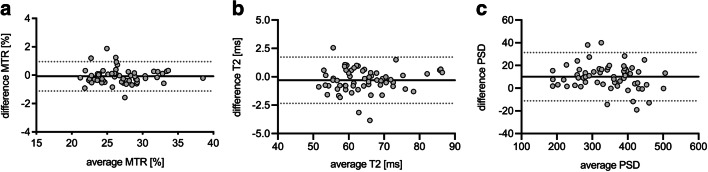
Bland-Altman plots for assessment of interreader reliability of magnetization transfer ratio (MTR) (**a**), transverse relaxation time T2 (**b**), and proton spin density (PSD) (**c**) of the sciatic nerve. The black continuous line represents the mean of all differences (bias), grey dotted lines show the 95% limits of agreement

Bland-Altmann plots for test-retest reliability are shown in Fig. 4 and for interreader reliability in Fig. 5. Bland-Altman analysis showed a rather random distribution of measurement error with low bias between raters and scans. Also, no proportional bias such as systematically higher measurement error for higher or lower MTR, T2, or PSD values could be observed. Overall mean differences between readers were 0.1% for MTR, 0.3 ms for T2, and 10.1 for PSD.

## Discussion

This study evaluates the interreader and test-retest reliability of quantitative MRN biomarkers like MTR, T2 relaxation time, and PSD of the sciatic nerve in a cohort of 21 healthy participants. Each participant underwent three MRN exams on separate days which were analyzed by two independent readers. By reporting ICCs, SEM, and MDD values, we quantify measurement imprecision of all readout parameters.

While T2 relaxometry and MTI are increasingly studied as quantitative MRN techniques, they have not been implemented in clinical routine yet [11–14, 24, 26]. Before interpreting MRN biomarkers in individual patients, assessment of reliability is required not only in qualitative categories but particularly by precise quantification of measurement error.

This study adds to the field by providing a systematic quantification of interreader and test-retest reliability in a larger cohort for both peripheral nerve MTI and T2 relaxometry biomarkers. With regard to peripheral nerve MTI, we are aware of two previous studies that have assessed reliability in smaller cohorts (Yiannakas et al [28]: 5 participants, ICC_interreader_ 0.65, ICC_test-retest_ 0.76; Dortch et al [11]: 13 participants, ICC_interreader_ 0.92, ICC_test-retest_ 0.69), although the main focus of these studies was on other research concerns [11, 28]. Regarding T2 relaxometry, Sollmann et al [29] have recently reported promising first results for interreader reliability while test-retest reliability had not been assessed yet to our knowledge.

While the ICC is a widely used index of reliability, it depends not only on the measurement error itself but also on the variance of the measured parameter in the examined cohort [38–40]. To provide a parameter of measurement imprecision that is independent of the variance of the biomarker in the test cohort, we here additionally report the SEM for all assessed biomarkers.

Moreover, the SEM allows for the calculation of the MDD, which indicates the difference in repeated measurements needed to attribute a measured difference to a change in the true value with a 95% certainty, but not to a fluctuation due to measurement error [31, 32]. The SEM and MDD are therefore of high significance when implementing quantitative biomarkers in clinical practice.

To value the clinical relevance of the measurement error of T2 relaxometry and MTI, the MDD can be regarded in context with results from previous studies in the setting of specific neuropathies and healthy controls as summarized in Tables 4 and 5.

**Table 4:** MTR values in health and disease as reported in previous studies of peripheral nerve imaging (non-exhaustive)

Authors	Examined pathology	Examined nerve	Healthy controls: [mean MTR ± SD in %]	Disease: [mean MTR ± SD in %]	Difference mean MTR in % (healthy – disease)
Dortch et al [11]	Charcot-Marie-Tooth types 1A and 2A	Sciatic nerve	37.2 ± 2.3 (n = 21)	33.8 ± 3.3 and 31.5 ± 1.9 (n = 10 and 3)	3.4 (1A) and 5.7 (2A)
Fortanier et al [12]	Charcot-Marie-Tooth types 1A	Sciatic and tibial nerve	39.5 ± 3^†^ (n = 13)	33.7 ± 3.7^†^ (n = 32)^†^	5.8
Kollmer et al [14]	Hereditary transthyretin amyloidosis	Sciatic nerve	39.4 ± 2.1 (n = 20)	26.4 ± 0.7^‡^ (n = 25)^‡^	13
Kollmer et al [24]	5q-linked spinal muscular atrophy (SMA 3)	Sciatic nerve	32.4 ± 0.6 (n = 18)	26.2 ± 0.7 (n = 18)	6.2
Kollmer et al [41]	Dependency of MTI on age and location, only healthy participants	Sciatic and tibial nerve	19.9 ± 1^*^ (n = 10)	-	-
Pridmore et al [42]	Hereditary neuropathy with liability to pressure palsies	Sciatic nerve	39.4 ± 2 (n = 7)	37.6 ± 3.8 (n = 10)	1.8

Mean values for magnetization transfer ratio (MTR) as described by previous studies for various diseases. ^†^Only sciatic nerve values are displayed, ^‡^values for patients with symptomatic polyneuropathy, ^*^values for the distal sciatic nerve in a younger age group (20–32 years). Underlined differences were considered significant. Measures of precision as the standard error of measurement (SEM) and the minimum detectable difference (MDD) may be interpreted in conjunction with these values

**Table 5:** Nerve transverse relaxation times T2 and/or proton spin density (PSD) values in various diseases as reported in previous studies of peripheral nerve imaging (non-exhaustive)

Authors	Examined pathology	Examined nerve	Healthy controls: [mean ± SD, T2 in ms]	Disease: [mean ± SD, T2 in ms]	Difference mean T2/PSD (healthy – disease)
Vaeggemose et al [4]	Type 1 diabetes with diabetic neuropathy	Sciatic nerve	T2: 79 ± 8 PSD: 381 ± 80 (n = 30)	T2: 83 ± 7^*^ PSD: 343 ± 77^*^ (n = 11)^*^	4 38
Fortanier et al [12]	Charcot-Marie-Tooth types 1A	Sciatic and tibial nerve	PSD: 490 ± 65 (n = 13)	PSD: 557 ± 89^†^ (n = 32)	67
Kollmer et al [13]	Amyloid light chain (AL) amyloidosis	Sciatic nerve and distal branches	T2: 75.4 ± 2 PSD: 310.5 ± 14.1 (n = 25)	T2: 102 ± 14.4^+^ PSD: 525.5 ± 53^+^ (n = 7)^+^	26.6 215
Jende et al [25]	Multiple sclerosis	Sciatic nerve and distal branches	T2: 82 ± 2.1 PSD: 266 ± 11 (n = 35)	T2: 64.3 ± 1 PSD: 371.8 ± 7.7 (n = 36)	17.7 105.8
Kronlage et al [26]	Chronic inflammatory demyelinating polyneuropathy	Sciatic nerve	T2: 70.3 ± 1.4 PSD: 159.7 ± 7.1 (n = 18)	T2: 72.3 ± 2.9 PSD: 177.2 ± 7.4 (n = 18)	2 17.5
Vaeggemose et al [27]	Charcot-Marie-Tooth types 1A	Sciatic and tibial nerve	T2: 79 PSD: 403 (n = 30)	T2: 81 PSD: 561 (n = 15)	2 158
Sollmann et al [29]	Lumbar disc herniation with nerve compression	Sciatic nerve	T2: 43.3 ± 2.4 (n = 21)	T2: 61.5 ± 6.2^‡^ (n = 5)	18.2
Kollmer et al [43]	Transthyretin familial amyloid polyneuropathy (TTR-FAP)	Sural nerve	T2: 79 ± 2.7 PSD: 258.2 ± 9.1 (n = 35)	T2: 89.4 ± 5.1^§^ PSD: 430 ± 15.3^§^ (n = 25)^§^	10.4 171.4
Kollmer et al [44]	Transthyretin familial amyloid polyneuropathy (TTR-FAP)	Sciatic nerve and distal branches	T2: 84.1 ± 2.5 PSD: 286.6 ± 10 (n = 40)	T2: 103.9 ± 6.4^§^ PSD: 550 ± 35.8^§^ (n = 13)^§^	19.8 263.4
Felisaz et al [45]	Chronic inflammatory demyelinating polyneuropathy	Tibial nerve	T2: 55.7 (range: 30–94) (n = 10)	T2: 52.8 (range: 32–81) (n = 10)	2.9
Pham et al [46]	Diabetic neuropathy	Sciatic nerve and distal branches	PSD: 288 ± 13.4 (n = 25)	PSD: 360 ± 22.9^#^ (n = 10)^#^	72

Mean values for T2 and proton spin density (PSD) as described by previous studies for various diseases. ^*^Values for patients with severe polyneuropathy, ^†^only sciatic nerve values are displayed, ^+^values for patients with moderate AL-polyneuropathy, ^‡^acquired using a T2-prepared turbo spin-echo sequence, ^§^values for patients with symptomatic TTR-FAP, ^#^values for patients with severe polyneuropathy. Underlined differences were considered significant. Measures of precision as the standard error of measurement (SEM) and the minimum detectable difference (MDD) may be interpreted in conjunction with these values

For MTR of the sciatic nerve, the current study implies an MDD value of 4.7%. In previous studies, significant differences in MTR between patients and healthy controls ranged from 3.4 to 13%, and in three out of four studies a difference of over 4.7% was observed (Table 4) [11, 12, 14, 24]. Notably, the mean MTR of our cohort was lower than in most of the control groups listed in Table 4. This most likely has technical reasons, since MTR values depend on various factors, such as magnetic field strength, coil characteristics, and sequence parameters, like offset frequency and power of the saturation pulses [47–50].

For T2 of the sciatic nerve, the present study implies an MDD of approximately 7.4 ms. For comparison, previously reported significant differences of T2 ranged from 10.8 to 26.6 ms between patients and control groups (Table 5) [13, 25, 29, 43, 44].

For PSD, our results imply an MDD of 55.7 (test-retest) and 59 (interreader). PSD is considered a semi-quantitative parameter that directly depends on the MR signal and associated parameters, such as receiver gain. Therefore, reliability values of PSD reported in this study may only be regarded as orientation values and in conjunction with the absolute mean values of other studies (Table 5). To overcome this limitation, we additionally normalized PSD values by using adjacent muscles. Although these normalized PSD values may serve as a more robust parameter when using different setups, they may however be influenced by muscular changes which can occur in the context of systemic neuropathies [51].

While the calculated MDDs are often smaller than the mean differences between patients and healthy controls, it is not clear whether changes in biomarkers in longitudinal follow-up studies will exceed these limits. So far, we are not aware of any longitudinal studies assessing MTR, T2, or PSD in the PNS. However, such longitudinal studies are crucial to further evaluate the potential of quantitative MRN in a clinical follow-up setting.

Although quantitative MRN offers multiple contrasts, neuropathies of different etiologies share non-specific changes as an increase in T2 or PSD and a decrease in MTR, which may limit their potential in differential diagnosis [52]. Furthermore, normative data may depend on scanning parameters, demography, and postprocessing methods [33, 41]. However, if reliability is proven, quantitative biomarkers could allow tracking disease progression or responsiveness under therapy. In order to implement quantitative MRN as a clinical tool, more studies on normative data should be carried out in the future.

Our study has limitations. First, we restricted the analysis to the sciatic nerve, since it is the most commonly examined nerve and well suited for MRN due to its large caliber and straight course and therefore most appropriate for MSE and GRE sequences with lower special resolution. An inclusion of smaller nerves or nerves with an oblique course may have led to different values of measurement error. Thus, all values presented should primarily be used for MRN of the sciatic nerve and may generally serve as orientation values under optimal conditions. Second, our cohort only consisted of healthy young participants and demographic variables were relatively homogeneous. This also allowed for assessment of measurement error under rather good conditions, since from our experience motion artifacts are less commonly observed in younger subjects. The examined cohort was male, however, we would not expect different results for female participants, since all parameters have been shown to not systematically differ between sexes [33, 41]. Furthermore, planning of the MR examination and image postprocessing were performed by experienced neuromuscular radiologists, and also for this reason the proposed orientational values should rather be regarded as minimum values. Besides, MTR may depend on B1 field inhomogeneities, which is why correction schemes may be applied to neuromuscular imaging [11, 12, 53]. To reduce the effects of B1 field inhomogeneities on the received signal, we applied an adaptive inline image filter and restricted analysis to six central slices where the B1 field is expected to be more homogeneous than at the edges of the slab. Lastly, this is a single-center, single-vendor study and measured parameters may depend on the hardware, sequence parameters, and postprocessing methods, limitations that are inherent to most quantitative imaging studies. While we measured test-retest reliability in a follow-up setting with one particular scanner, we would expect higher measurement error if multiple scanners were used, as differences in amplification of the MR signal modify PSD and differences in saturation pulses may affect MTR.

In conclusion, this study demonstrates that MTI and T2 relaxometry of the sciatic nerve results in reliable and reproducible values. By assessing the SEM for all examined parameters, it provides quantitative data to measure the imprecision that is associated with multiple scans or different observers. These values may be considered as orientation values of measurement error in further studies and potential clinical applications of quantitative MRN.

## Supplementary Information

ESM 1 (DOCX 880 kb)

## Abbreviations

DTI: Diffusion tensor imaging
ICC: Intraclass correlation coefficient
MDD: Minimum detectable difference
MRN: MR neurography
MTI: Magnetization transfer imaging
MTR: Magnetization transfer ratio
PNS: Peripheral nervous system
PSD: Proton spin density
SEM: Standard error of measurement

## Declarations

### Guarantor

The scientific guarantor of this publication is Moritz Kronlage.

### Statistics and Biometry

Rouven Behnisch (Institute of Medical Biometry and Informatics, University of Heidelberg) conducted the statistical analysis and is co-author of this manuscript.

### Study subjects or cohorts overlap

Several subjects of this study also participated in another study on diffusion tensor imaging (DTI): Preisner F, Bäumer P, Wehrstein M et al (2019) Peripheral Nerve Diffusion Tensor Imaging : Interreader and Test-retest Reliability as Quantified by the Standard Error of Measurement. Clin Neuroradiol. 10.1007/s00062-019-00859-0.

Apart from the demographic variables, there is no overlap especially not in results or conclusions as all data concerning MTI and T2-Relaxometry have not been analyzed before.

### Methodology

• prospective study

• observational

• performed at one institution
