# Supplementary material for: Reliability and reproducibility of sciatic nerve magnetization transfer imaging and T2 relaxometry
Source: Eur Radiol. 2021 Jun 9;31(12):9120–30. doi: 10.1007/s00330-021-08072-9 (PMC8589742; doi:10.1007/s00330-021-08072-9)

**Supplementary Figure 1.** Positioning of the field of view (FOV) for all imaging sequences. Care was taken that the FOV for MTI- and MSE-sequences was corresponding to the distal end of the M. rectus femoris muscle belly (*), as indicated on the sagittal localizer.


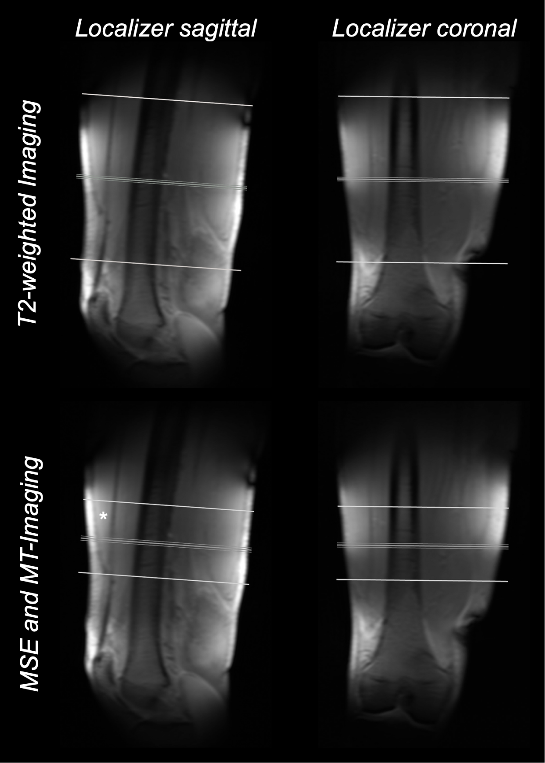


**Supplementary Figure 2.** Additional scatterplots to visualize readout parameters of all participants, scans and readings. MTR = magnetization transfer ratio, T2 = transverse relaxation time, PSD = proton spin density.


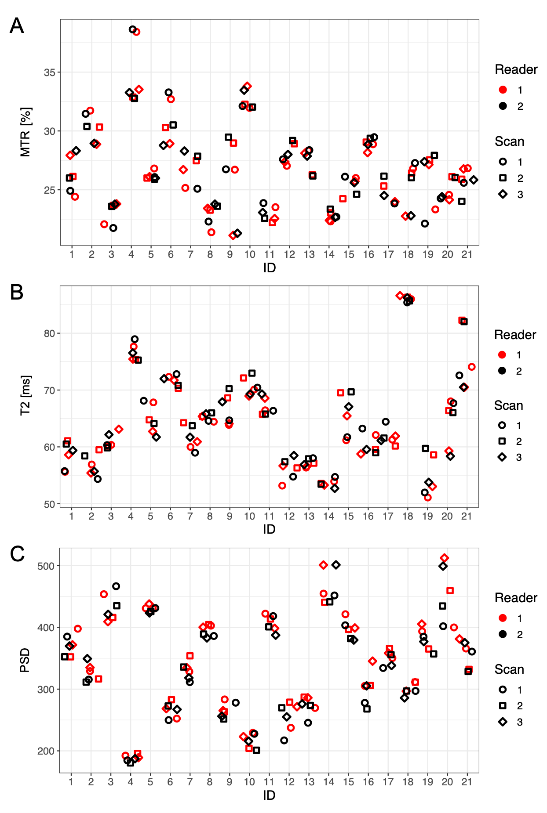


**Supplementary Figure 3.** Exemplary MTR- and T2-maps of the sciatic nerve. MTR-maps were computed with Python (Version 3.7.3, Continuum Analytics) using the Pydicom (Version 2.1.2) and Numpy (Version 1.17.3) packages. T2-maps were calculated using the OsiriX plug-in T2 Fit Map. The arrow points towards the tibial portion of the sciatic nerve. MTR = magnetization transfer ratio, TSE = turbo spin echo.


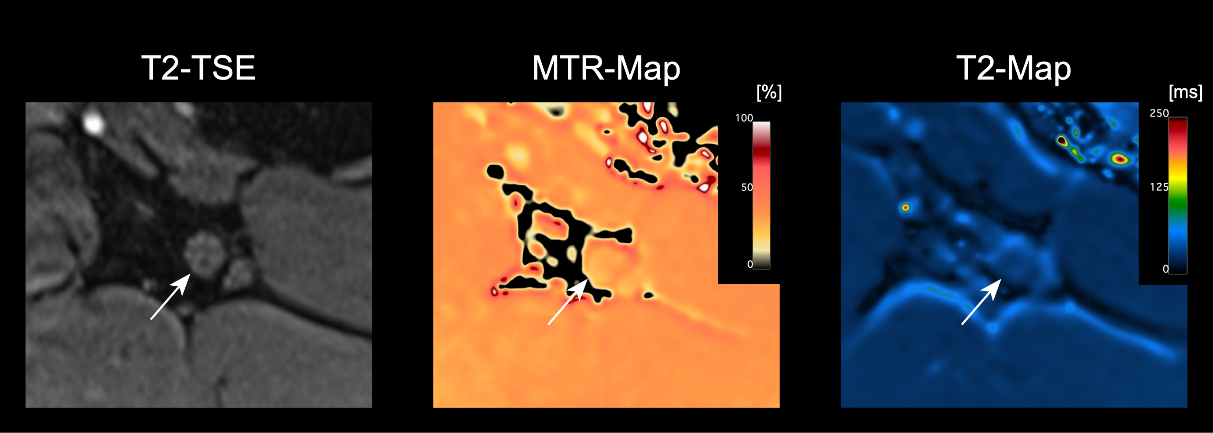

Supplement: Supplementary file 1 — (DOCX 880 kb) [file 330_2021_8072_MOESM1_ESM.docx]
